# Adsorption of Pb(II) and Cu(II) by Ginkgo-Leaf-Derived Biochar Produced under Various Carbonization Temperatures and Times

**DOI:** 10.3390/ijerph14121528

**Published:** 2017-12-07

**Authors:** Myoung-Eun Lee, Jin Hee Park, Jae Woo Chung

**Affiliations:** 1 Department of Urban System Engineering, Gyeoungnam National University of Science and Technology (GNTECH), Dongjin-ro 33, Jinju, Gyeongnam 52725, Korea; myoung22eun@naver.com; 2 Department of Environmental and Biological Chemistry, Chungbuk National University, Cheongju, Chungbuk 28644, Korea; pjinh@chungbuk.ac.kr; 3 Department of Environmental Engineering, Gyeoungnam National University of Science and Technology (GNTECH), Dongjin-ro 33, Jinju, Gyeongnam 52725, Korea

**Keywords:** ginkgo-leaf-derived biochar, carbonization condition, adsorption, lead, copper

## Abstract

Ginkgo trees are common street trees in Korea, and the large amounts of leaves that fall onto the streets annually need to be cleaned and treated. Therefore, fallen gingko leaves have been used as a raw material to produce biochar for the removal of heavy metals from solutions. Gingko-leaf-derived biochar was produced under various carbonization temperatures and times. This study evaluated the physicochemical properties and adsorption characteristics of gingko-leaf-derived biochar samples produced under different carbonization conditions regarding Pb(II) and Cu(II). The biochar samples that were produced at 800 °C for 90 and 120 min contained the highest oxygen- and nitrogen-substituted carbons, which might contribute to a high metal-adsorption rate. The intensity of the phosphate bond was increased with the increasing of the carbonization temperature up to 800 °C and after 90 min of carbonization. The Pb(II) and Cu(II) adsorption capacities were the highest when the gingko-leaf-derived biochar was produced at 800 °C, and the removal rates were 99.2% and 34.2%, respectively. The highest removal rate was achieved when the intensity of the phosphate functional group in the biochar was the highest. Therefore, the gingko-leaf-derived biochar produced at 800 °C for 90 min can be used as an effective bio-adsorbent in the removal of metals from solutions.

## 1. Introduction

The production of renewable energy and bioproducts from nonfood biomass is a sustainable strategy to address the worldwide energy and climate-change challenges [1,2]. Biochar is a product of the thermal decomposition of biomass under a partial or total absence of oxygen gas (O_2_), and it has been recently proposed as a soil amendment to sequester the carbon (C) in soils for which a C-negative process is used, as well as for the prevention of greenhouse gas emissions and soil quality improvements [3,4,5,6]. Furthermore, biochar can reduce the mobility of contaminants [7].

Environmental remediation has been recently recognized as a promising area in which biochar can be successfully applied [8,9]. Choi et al. tested the heavy metal (lead (Pb), copper (Cu), cadmium (Cd), and zinc (Zn)) sorption capacity of a biochar produced from a sesame seed byproduct in metal-contaminated wastewater [10]. Xu et al. applied a rice husk biochar and a dairy manure biochar as sorbents for the simultaneous removal of Pb, Cu, Cd, and Zn from aqueous solutions [11]. Chen et al. evaluated the adsorption characteristics of a hardwood biochar that was obtained at 450 and 600 °C for the removal of Cu and Zn in an aqueous solution [12]. Tong et al. showed that biochar prepared from the straws of peanut (*Arachis hypogaea*), soybean (*Glycine max*), and canola (*Brassica napus*) adsorbed Cu through the formation of surface complexes [13]. The maximum Cu sorption capacity of these biochar varieties was greater than that of commercial activated C (AC). In the authors’ previous study, a peat moss (*Sphagnum*)-derived biochar showed a maximum adsorption capacity of 81.3 mg/g for Pb, 18.2 mg/g for Cu, and 39.8 mg/g for Cd, which is much higher than the adsorption capacity of commercial AC [14].

A variety of waste biomasses such as crop residues, wood, olive waste, peat moss, forestry waste, food processing waste, paper mill waste, manure, and sewage sludge have been converted into biochar for the provision of a sorbent for the removal of contaminants from wastewater [14,15,16,17,18]. Singh et al. used carbonized electrospun nanofibrous membranes (CNMs) to remove disinfection byproducts and found CNMs to have a chloroform adsorption capacity of 554 mg/g [19]. The use of biochar for the removal of disinfection byproducts can be another challenge. The choice of an appropriate biomass is very important in terms of the environmental applications of biochar. 

Ginkgo trees are widely used as street trees because the species is not only robust against both hot and cold climatic conditions, but it is also resistant to pollution and insect pests. As of 2015, 6,784,210 street trees exist in Korea, and 1,004,801 (14.8%) are ginkgo trees. In Seoul, the capital city of Korea, 117,556 of the 303,144 street trees are ginkgo trees, which account for approximately 39% of the total number of street trees. Annually, large amounts of fallen leaves are found on the streets that need to be cleaned and treated. Currently, most of the fallen leaves are being treated using landfills or incineration, while some portions are being recycled as compost or livestock feed; therefore, it is very important to find suitable recycling methods for the fallen tree leaves. Accordingly, for this study, gingko leaves (GLs) were selected as the raw material of the metal-adsorbent biochar production.

The objective of this study is to evaluate the physicochemical properties and adsorption characteristics of gingko leaf-derived biochars (GBs) that have been produced under different carbonization conditions regarding Pb(II) and Cu(II).

## 2. Materials and Methods 

### 2.1. Preparation of the Biochars

Fallen GLs were collected from the streets in autumn, washed with water, and then dried in a drying oven at 105 °C. A 50-mL capacity crucible was filled with 5–10 g of the GLs, and the crucible contents were then purged with nitrogen gas (N_2_) for 5 min. The GBs were produced under various pyrolysis temperatures (400, 600, 800, and 1000 °C) and for various times (1, 2, 5, 10, 30, 60, 90, 120, 180 min) in an electric furnace. In the authors’ previous study, the optimal carbonization temperature and time for the heavy metal adsorption of the peat moss-derived biochar were 800 °C and 90 min, respectively [14]; therefore, for the carbonization temperature experiment of the present study, the carbonization time was set as 90 min. After the determination of the optimum temperature, the biochar was prepared by carbonization for 1, 2, 5, 10, 30, 60, 90, 120, and 180 min under the optimal temperature conditions to find the optimal carbonization time. The prepared biochar was ground and sieved (<2 mm) by passing it through a 32-mesh sieve. The biochar yield was calculated according to Equation (1), as follows:

(1) $$\mathrm{Biochar}\;\mathrm{yield}\;\left(\%\right)\;=\;\frac{weight\;of\;biochar}{weight\;of\;ginkgo\;leaf}\;\times \;100$$

### 2.2. Characterization of the Produced Biochars

To characterize the biochars that were produced at different carbonization temperatures and times, the elemental compositions, pH values, and functional groups were analyzed. The elemental compositions of the biochars were analyzed using a Flash 2000 Series elemental analyzer (Thermo-Fisher Scientific, Waltham, MA, USA). The ash contents were determined from the combustion of the dry samples at 760 °C for 6 h [20,21]. For the pH measurement, 5 g of the biochar was mixed with 25 mL of distilled water in a 50-mL tube for 1 h. The functional groups on the biochar surface were identified using a Spectrum One Fourier transform infrared (FTIR) spectroscopy system (Perkin Elmer, Foster City, CA, USA).

### 2.3. Metal Adsorption Experiments

To investigate the heavy metal adsorption characteristics of the GBs, experiments were conducted on the Pb(II) and Cu(II) adsorption rates. All of the chemicals and reagents that were used were of analytical grade. For the preparation of the Pb(II) and Cu(II) stock solutions (1000 mg/L), lead(II) nitrate (Pb[NO_3_]_2_) and copper(II) nitrate (Cu[NO_3_]_2_) were dissolved in Milli-Q water. The working solutions were prepared by the dilution of the stock solutions to specific concentrations. To determine the optimum carbonization temperature, the adsorption experiments of the biochars that were prepared at different temperatures were carried out using initial Pb(II) and Cu(II) concentrations of 60 mg/L. After the optimum temperature was determined, the adsorption experiments of the biochars prepared at different carbonization times were carried out using the initial Pb(II) and Cu(II) concentrations of 100 mg/L. All of the experiments were conducted at 25 °C. To prevent pH variation during the adsorption, the heavy metal solution was prepared using 0.07 M sodium acetate (NaOAc) and 0.03 M acetic acid (CH_3_COOH) at a pH of 5 ± 0.1 [22]. Experiments were performed in triplicate and the results were presented as means ± standard deviations of the triplicates. 

## 3. Results

### 3.1. Elemental Compositions and pH of the Ginkgo-Leaf-Derived Biochars

The elemental compositions of the GLs and the GBs that were produced under different carbonization temperatures are shown in Table 1. The biochar pH increased with the increasing of the carbonization temperature in the range of 10.2–12.9. The C content of the biochar decreased with the increasing of the carbonization temperature. The hydrogen (H) content was the highest at 600 °C. The nitrogen (N) and oxygen (O) contents increased with the increasing of the carbonization temperature up to 800 °C, after which they decreased.

Table 2 shows the elemental compositions of the GLs and the GL-derived biochars that were produced under different carbonization times. The elemental concentrations did not show consistent decreases or increases with the increasing of the carbonization time. Generally, the carbon content was the lowest between 90 and 120 min of carbonization, and the N and S contents were the highest. Therefore, the biochars that were produced at 90 and 120 min contained N and S substituted carbon functional groups with a high reactivity.

### 3.2. Functional Groups of the Ginkgo-Leaf-Derived Biochars

**Figure 1:**
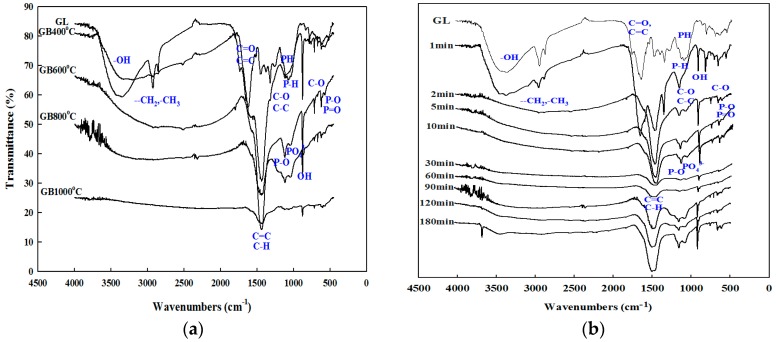
Fourier-transform-infrared (FTIR) spectra of the ginkgo leaf (GL) and the ginkgo leaf-derived biochars (GBs) that were produced under different carbonization temperatures (**a**) and times (**b**).

The functional groups of the biochars that were produced under different carbonization conditions were investigated, as shown in Figure 1. Various functional groups including hydroxyl (-OH), ethylene (-CH_2_), methyl (-CH_3_), carbonyl (C=O), and dicarbon (C=C) were found in the GLs. The GL functional groups disappeared in the GBs as the carbonization temperature was increased. In particular, the functional groups in the region higher than 1600 cm^−1^ disappeared with the heating application. As the carbonization time was increased, the amount of the O functional groups from the GLs increased in the first 1–2 min. Afterward, the H-bonded OH groups, aliphatic CH, C-H_2_, C-H_3_ stretching, and C=O stretching of the carboxylic acid disappeared with the increasing of the carbonization time. Along with the disappearance of functional groups of the carboxylic acid such as the H-bonded OH groups, aliphatic CH, C-H_2_, C-H_3_ stretching, and C=O stretching, the intensities of the functional groups of the aliphatic-CH deformation from 1420–1470 cm^−1^, the H-bonded OH stretching from 750–880 cm^−1^, and the P-O stretching at 1114 cm^−1^ increased with the increasing of the carbonization time after 90 min.

### 3.3. Lead(II) and Cu(II) Adsorption Characteristics of the Biochars

Table 3 shows the influences of the carbonization temperature on the biochar yield and the Pb(II) and Cu(II) adsorption capacities. The biochar yield decreased with the increasing of the carbonization temperature. The Pb(II) and Cu(II) adsorption capacities were the highest for the GB that was produced at 800 °C among the values tested. 

The effects of the carbonization time on the biochar yield and its Pb(II) and Cu(II) adsorption capacities are shown in Table 4. The biochar yield decreased with the increasing of the carbonization time. The Pb(II) and Cu(II) adsorption capacities of the biochars increased with the increasing of the carbonization time. The GB that was produced at 90 min showed the highest Pb(II) and Cu(II) adsorption capacities, which then decreased again after 120 min.

## 4. Discussion

The carbonization significantly increased the pH level of the GLs from 4.6 to 10.2–12.9. The increase of the pH is attributed to the formation of alkali groups in the biochar as well as functional groups such as -carboxylate (COO^−^) and -O^−^ in the biochar that are caused by the carbonization process [23]. A similar decrease of the C content with the increasing of the carbonization temperature was reported in the carbonization of the rice husk and the rice straw, but this was not observed in the biochar that was derived from wood because of the relatively higher aromatic organic matter of raw materials [24]. The degree of the carbonization in the biochar production increased with the increasing of the temperature in previous studies [25,26]. To characterize the relationships between the carbonization temperature and the degrees of the aromaticity and the hydrophobicity of the biochars, the molar ratios of C, H, N, and O were calculated, as shown in Table 1. The combustion-condensate particulates, charcoal, and char can be divided based on the O/C molar ratio of 0:2. Condensate particulates do not comprise the relic structures of the original biomass, whereas charcoal and char contain the relic structures of raw materials [27]. The O/C ratio of the biochar that was produced at 1000 °C was smaller than 0:2, and therefore it can be grouped into the condensate particulates and the biochars that were produced at less than 800 °C that, in turn, could be classified as combustion residues. The decrease of the H/C value of the biochar compared with that of the GLs indicates increases of the aromatization and carbonization of all of the investigated materials [21]. The lowest H/C ratio (0.091) at 1000 °C suggests that the biochar is highly carbonized, thereby indicating a higher aromaticity at 1000 °C compared with the other temperatures investigated. The highest O/C and (O + N)/C ratios of the biochar that were produced at 800 °C indicate a structural rearrangement of the aromatic rings and a substitution by the N and O functional groups, which increases the black-carbon reactivity [6].

The adsorption of the heavy metals on the surface of the biochar occurred due to the involvement of the functional groups. The disappearance of functional groups in the region higher than 1600 cm^−1^ is the result of the destruction of the cellulose and the lignin in the feedstocks, which is due to the high temperature during the pyrolysis process. Refig et al. showed that the O-H stretch peak at the approximate region from 3600–3200 cm^−1^ was clearly visible in a char that had been produced at 300 °C, but it was decreased as the pyrolysis temperature was increased, thereby representing the dehydration of the cellulose and ligneous compounds [27]. Gai et al. also showed that the thermal destruction of the cellulose and the lignin in the feedstocks exposed functional groups such as hydroxyl -OH, ester-C=O, aliphatic alkyl-CH_2_-, and aromatic-C=O in the biochars [28]. 

H-bonded OH groups from 3300–3670 cm^−1^, aliphatic CH, C-H_2_, C-H_3_ stretching from 2850–2950 cm^−1^, carboxylic-acid C=O stretching from 1585–1640 cm^−1^, C=O stretching of the cyclic and acyclic double-bond from 1585–1640 cm^−1^ [29], and carboxylic-acid salt from 1332–1390 cm^−1^ were found in the GL. As the carbonization temperature was increased, the functional groups such as the H-bonded OH groups, aliphatic C-H, C-H_2_, C-H_3_ stretching, and carboxylic-acid C=O stretching were extinguished. In addition, the functional groups such as the aliphatic-CH deformation from 1420–1470 cm^−1^ and the H-bonded OH stretching from 750–880 cm^−1^ were produced. The H-bonded OH stretching and the C-O stretching disappeared at 1000 °C. The band at 1626 cm^−1^ was attributable to the C=C, C=O, and aromatic C=C that were reduced at the carbonation temperature of 1000 °C. 

The band at 1090 cm^−^^1^ is the P-O stretching vibrations of PO_4_^3−^ [30]. In the mid-infrared region under 1300 cm^−1^, where most of the P-bond vibrations can be detected, tricalcium phosphate revealed peaks at 1114 cm^−1^ that correspond to the P-O stretching vibration [31]. Regarding the P-O stretching, dicalcium phosphate and dicalcium phosphate dihydrate share common peaks in the approximate regions of 1130 and 990 cm^−1^ [31], and at 1066 cm^−1^; this is also the case regarding the ionized-linkage P^+^-O^−^ in the acid-phosphate esters [32]. The spectrum of iron(III) phosphate dehydrate is dominated by two broad peaks at 1037 cm^−1^, regarding the PO_4_^3−^ stretching, and 541 cm^−1^, regarding the P-O or P=O stretching [30]. The peak at 1040 cm^−1^ (PO_4_^3−^or P-H deformation) was only visible in the spectrum of the amorphous calcium phosphate [31,33]. The intensity of the phosphate (PO_4_^3−^) bonds was increased with the increasing of the carbonization temperature, but it disappeared in the GB that was produced at 1000 °C. 

The decrease of biochar yield with the increasing of the carbonization temperature was due to the volatilization of organic matters by heating. When the temperature is higher than 302 °C, the cellulose depolymerizes and produces volatile compounds [1]. The dehydration of the hydroxyl groups and ligno-cellulose decomposition during the pyrolysis process of a biochar reduced the biochar yield [26,34]. The reductions of the yield and the volatile-matter content are reportedly caused by the decomposition of the organic matter and the loss of methane (CH_4_), H_2_, and carbon monoxide (CO) with the increasing of the carbonization temperature [25,26], which was also confirmed by the FTIR spectra.

The highest heavy metal adsorption capacity of the GB produced at 800 °C can be explained by the FTIR results. The highest intensity of the PO_4_^3−^ functional group in the FTIR spectra might result in the highest heavy metal adsorption capacities at 800 °C. In the case of the Pb(II) adsorption, 99.2% of the added Pb was removed by the GB that was produced at 800 °C, while the Cu(II) removal rate was 34.2%. The higher Pb(II) adsorption is attributed to the formation of a stable compound of Pb(II) and phosphorous (P) on the biochar surface and the higher affinity of Pb(II) compared with that of Cu(II). The sorption of metal ions from an aqueous solution is generally governed by surface chemistry and the surface area of the sorbent, or by the precipitation reactions [35]. Higher Pb removal rate of a biochar may be attributed to the precipitation of Pb as PO_4_^3−^ and carbonate (CO_3_^2−^) compounds [11,36]. Phosphate forms Pb pyromorphite (Pb_5_ [PO_4_]_3_ X; X = Cl^−^, OH^−^, F^−^, Br^−^, etc.), which exhibits a very low solubility [9,37,38].

The biochar yield decreased with the increasing of the carbonization time (Table 4). Gheorghe et al. showed that the char yield decreased as the carbonization time was increased from 5–30 min at 450 °C [39]. Lee et al. also showed that the biochar yield decreased with the carbonization time from 30–90 min at 400–1000 °C [14]. Increasing weight loss with increasing carbonization temperature and time was attributed to the increasing amount of combusted organic contents [14]. The decline in biochar yield with increasing temperature can be explained by the thermal degradation of ligno-cellulose structures and the dehydration of hydroxyl groups [19]. The highest adsorption of the Pb(II) by the biochar produced at 90 min can be attributed to the highest PO_4_^3−^-peak intensity in the FTIR spectra. In our previous studies, a peat moss-derived biochar that had been produced at 800 °C for 90 min showed the highest heavy metal adsorption capacity in a solution [14,40]. Therefore, in terms of metal adsorption, the biochar that was produced at 800 °C for 90 min was the most effective adsorbent for the removal of heavy metals from wastewater.

## 5. Conclusions

The GB that was produced at 800 °C for 90 min was the most efficient for the adsorption of Pb(II) and Cu(II) in an aqueous solution, which might be attributed to a well-developed porous structure and the PO_4_^3−^ functional group, respectively, both of which were higher than those of the GLs. Due to the existence of the highest intensity of the PO_4_^3−^ functional group in the FTIR spectra, the highest adsorption of heavy metals might occur at 800 °C. The higher Pb(II) adsorption is attributed to the formation of a stable compound of Pb(II) and P on the surface of the biochar, in addition to the higher affinity of Pb(II) compared with Cu(II).

## Figures and Tables

**Table 1:** Elemental composition of the ginkgo leaf (GL) and the ginkgo-leaf-derived biochars (GBs) produced under different carbonization temperatures.

Sample	Carbonization Temperature (°C)	Elemental Composition (%)						Atomic Ratio			pH
		C	H	N	S	O	Ash	O/C	H/C	(O + N)/C	
GL		45.14	5.54	1.21	0.73	33.99	13.40	0.565	1.473	0.588	4.60
GB	400	51.15	2.07	1.37	1.02	16.38	28.02	0.240	0.486	0.263	10.19
	600	48.14	3.81	1.29	0.87	17.31	32.63	0.270	0.950	0.293	10.72
	800	42.48	0.47	1.65	1.85	17.96	35.60	0.317	0.133	0.350	12.43
	1000	43.34	0.33	0.78	2.19	6.81	46.56	0.118	0.091	0.133	12.90

**Table 2:** Elemental composition of the ginkgo-leaf-derived biochars (GBs) produced under different carbonization times.

Elemental Composition (%)	Carbonization Time (min)								
	1	2	5	10	30	60	90	120	180
C	51.19	44.55	41.94	40.33	48.30	45.84	40.51	39.31	23.32
H	1.09	1.061	0.92	0.85	1.30	1.31	1.13	1.27	1.19
N	1.32	1.08	1.05	1.09	1.28	1.25	1.55	1.26	1.26
S	1.08	1.01	1.04	1.17	1.64	1.70	1.77	1.79	0.59
O	14.02	17.05	18.54	19.37	11.93	14.21	19.44	16.09	19.33
Ash	31.31	35.26	36.52	37.18	35.55	35.69	35.6	40.29	54.31

**Table 3:** Biochar yield and heavy metal adsorption capacities according to carbonization temperature. Data are the means ± standard deviation. Means with the same letter in a column do not significantly differ at *p* < 0.05, as determined by Duncan’s multiple range test with SPSS version 24 (IBM, Armonk, NY, USA). The high mean to low mean sequence is marked by the letters ^a^ to ^d^.

Carbonization Temperature (°C)	Biochar Yield (%)	Equilibrium Adsorption Capacity (mg/g)	
		Pb(II)	Cu(II)
Control (GL)	-	24.81 ± 0.19 ^d^	11.71 ± 0.35 ^c^
400	34.63 ± 2.28 ^a^	51.13 ± 0.42 ^b^	14.28 ± 0.32 ^b^
600	29.47 ± 1.21 ^b^	56.53 ± 0.00 ^a^	9.88 ± 0.11 ^d^
800	26.90 ± 0.89 ^b^	57.55 ± 0.02 ^a^	18.22 ± 1.00 ^a^
1000	20.62 ± 3.88 ^c^	42.89 ± 2.76 ^c^	11.03 ± 0.72 ^c,d^

**Table 4:** Biochar yield and heavy metal adsorption capacities according to carbonization time. Data are the means ± standard deviation. Means with the same letter in a column do not significantly differ at *p* < 0.05, as determined by Duncan’s multiple range test with SPSS version 24 (IBM, Armonk, NY, USA). The high mean to low mean sequence is marked by the letters ^a^ to ^f^.

Carbonization Time (min)	Biochar Yield (%)	Equilibrium Adsorption Capacity (mg/g)	
		Pb(II)	Cu(II)
Control (GL)	-	39.17 ± 0.74 ^f^	18.50 ± 0.71 ^b,c^
1	44.50 ± 0.37 ^a^	67.71 ± 1.30 ^d^	17.89 ± 1.09 ^c^
2	35.54 ± 0.46 ^b^	63.06 ± 3.53 ^e^	10.58 ± 1.52 ^e^
5	31.38 ± 0.65 ^c^	67.51 ± 0.61 ^d^	15.46 ± 1.99 ^d^
10	28.77 ± 0.44 ^d,e^	74.1 ± 0.31 ^c^	19.37 ± 0.57 ^b,c^
30	27.47 ± 0.12 ^e^	60.03 ± 1.89 ^e^	14.41 ± 0.75 ^d^
60	30.31 ± 0.16 ^c,d^	73.22 ± 1.86 ^c^	17.83 ± 0.18 ^c^
90	26.90 ± 0.89 ^e^	93.22 ± 1.63 ^a^	22.58 ± 0.20 ^a^
120	26.90 ± 0.31 ^e^	88.28 ± 0.26 ^b^	21.25 ± 0.64 ^b^
180	19.30 ± 2.96 ^f^	85.37 ± 0.52 ^b^	20.02 ± 0.28 ^b^
